# Supplementary material for: Impact of water, sanitation, and hygiene (WASH) interventions on gender-specific school attendance and learning outcomes: A systematic review and meta-analysis protocol
Source: PLoS One. 2024 Aug 1;19(8):e0308144. doi: 10.1371/journal.pone.0308144 (PMC11293655; doi:10.1371/journal.pone.0308144)
Supplement: S1 Data — (DOCX) [file pone.0308144.s002.docx]

**DATA EXTRACTION FORM USED IN THIS SYSTEMATIC REVIEW AND META-ANALYSIS**

| **Section** | **Field** | **Reviewer 1** | **Reviewer 2** |
| --- | --- | --- | --- |
| **Review Information** | Extraction Reviewer |  |  |
| **Study Identification** | ID |  |  |
|  | Authors |  |  |
|  | Title |  |  |
|  | Year of Publication |  |  |
|  | Country |  |  |
|  | Study Setting (Urban/Rural) |  |  |
|  | Type of School (Public/Private) |  |  |
| **Study Characteristics** | Study design |  |  |
|  | Sample Size |  |  |
|  | Response Rate |  |  |
| **WASH Availability and Attendance** | WASH available and students attend school (number of students) |  |  |
|  | WASH available and students do not attend school (number of students) |  |  |
|  | WASH not available and students attend school (number of students) |  |  |
|  | WASH not available and students do not attend school (number of students) |  |  |
| **Academic Performance and Learning Outcomes** | Test score |  |  |
|  | Grades |  |  |
| **Gender-Specific Data on Academic Performance and Learning Outcomes** | Boys attending school with WASH facilities (number of students) |  |  |
|  | Girls attending school with WASH facilities (number of students) |  |  |
|  | Boys not attending school despite WASH facilities (number of students) |  |  |
|  | Girls not attending school despite WASH facilities (number of students) |  |  |
|  | Boys' test scores |  |  |
|  | Girls' test scores |  |  |
|  | Boys' grades |  |  |
|  | Girls' grades |  |  |
