# Supplementary material for: Impact of water, sanitation, and hygiene (WASH) interventions on gender-specific school attendance and learning outcomes: A systematic review and meta-analysis protocol
Source: PLoS One. 2024 Aug 1;19(8):e0308144. doi: 10.1371/journal.pone.0308144 (PMC11293655; doi:10.1371/journal.pone.0308144)
Supplement: S1 File — (DOCX) [file pone.0308144.s003.docx]

**Identification of studies via other methods**

**Identification of studies via databases and registries**

Records Identified via Other sources (n =324 )

Records removed prior to screening

Duplicate records removed (n= )

Records still awaiting screening ( n= )

Records Identified from:

PubMed (n = 1677)

PubMed using free text (n =14)

Web of Science (n = 27)

Scopus (n = 7 )

Cochrane (n = 67)

**Identification**

Records screened (n = )

Records excluded (n = )

Reports not retrieved

(n = )

Reports sought for retrieval

(n = )

Reports sought for retrieval

(n = )

Reports not retrieved (n = )

**Screening**

Reports assessed for eligibility

(n = )

Reports excluded (n = )

Reports assessed for eligibility

(n = )

Reports excluded:

Studies included in review (n = )

Reports of included studies (n = )

**Included**

Figure1: PRISMA Diagram for the study selection (Page et al., 2021)
