# Supplementary material for: Impact of water, sanitation, and hygiene (WASH) interventions on gender-specific school attendance and learning outcomes: A systematic review and meta-analysis protocol
Source: PLoS One. 2024 Aug 1;19(8):e0308144. doi: 10.1371/journal.pone.0308144 (PMC11293655; doi:10.1371/journal.pone.0308144)
Supplement: S2 File — (DOCX) [file pone.0308144.s004.docx]

**Search Strategies used in this systematic review and Meta-Analysis**

**Database searches**

1. **PubMed Latest date of search: May 03/2024 at 06:15 PM**

**Free text search = total retrieved records = 14**

((((impact [tiab]) OR (effect [tiab])) AND ((((water [tiab]) AND (sanitation [tiab])) AND (hygiene [tiab])) OR (WASH [tiab]))) AND (School [tiab])) AND (attendance [tiab])

**Both free text and mesh term search =total retrieved record = 1677,**

((((((((("impact"[Title/Abstract] OR "effect"[Title/Abstract]) AND "water"[Title/Abstract]) OR "water"[MeSH Terms]) AND "sanitation"[Title/Abstract]) OR "sanitation"[MeSH Terms]) AND "hygiene"[Title/Abstract]) OR "hygiene"[MeSH Terms]) AND "School"[Title/Abstract]) OR "schools"[MeSH Terms]) AND "attendance"[Title/Abstract]

1. **Web of Science: Latest date of search: May 04/2024 at 11:24 AM**

**Web of Science using Boolean operators and nested queries = Total Retrieved record = 27**

TS=((" water, sanitation, and hygiene" OR " WASH interventions" OR " sanitation facilities" OR "impact of hygiene facilities") AND (" school attendance" OR "academic performance" OR "learning outcomes"))

1. **Scopus:** **Latest date of search: May 04/2024 at 3:10 PM; Total Retrieved record = 7**

TITLE-ABS-KEY ( "Impact of water, sanitation, and hygiene" OR "effect of water, sanitation, and hygiene" OR "effect of WASH" OR "impact of WASH" OR "effect of sanitation facilities" OR "impact of sanitation facilities" OR "effect of hygiene" OR "impact of hygiene" ) AND ( "school attendance" OR "academic performance" OR "learning outcomes" )

1. Cochrane Library: **Latest date of search: May 04/2024 at 11:24 AM;**

**Total Retrieved record = 67**

("water, sanitation, and hygiene" OR "WASH interventions" OR "sanitation" OR "hygiene") AND ("school attendance" OR "learning outcomes" OR "academic achievement")

**Other sources: Total Retrieved record = 324**

Additionally data from different search results were included in this systematic review and meta-analysis.
