# Supplementary material for: Impact of water, sanitation, and hygiene (WASH) interventions on gender-specific school attendance and learning outcomes: A systematic review and meta-analysis protocol
Source: PLoS One. 2024 Aug 1;19(8):e0308144. doi: 10.1371/journal.pone.0308144 (PMC11293655; doi:10.1371/journal.pone.0308144)
Supplement: Attachment — Submitted filename: PONE-D-24-18212_reviewer N.pdf [file pone.0308144.s005.pdf]

PLOS ONE

Impact of Water, Sanitation, and Hygiene (WASH) Interventions on Gender-Specific School Attendance and Learning Outcomes: A Systematic Review and Meta-Analysis Protocol

--Manuscript Draft--

No need to mention protocol word , The title already mentions "Systematic Review and Meta-Analysis," which implies a protocol is being followed.

**Manuscript Number:**	PONE-D-24-18212
**Article Type:**	Study Protocol
**Full Title:**	Impact of Water, Sanitation, and Hygiene (WASH) Interventions on Gender-Specific School Attendance and Learning Outcomes: A Systematic Review and Meta-Analysis Protocol
**Short Title:**	Impact of WASH Interventions on Gender-Specific School Attendance and Learning Outcomes protocol
**Corresponding Author:**	Dinaol Bedada Dibaba<br>Addis Ababa University<br>Addis Ababa, ETHIOPIA
**Keywords:**	WASH interventions, educational outcomes, school attendance.
**Abstract:**<br><br>Methodology and Result part usually in past tense, please read it again	The Water, Sanitation, and Hygiene (WASH) interventions have been acknowledged for their role in the public health and educational outcomes. While there are strong evidences that reveal that WASH facilities do reduce the prevalence of infectious diseases and improve the learning environment, data remain thin and equivocal on the differential impacts of WASH facilities on education by gender. The literature reviewed does not, in most cases; go to the extent of investigating if indeed both men and women students have unique needs especially in underprivileged areas. This is the point from which the present systematic review and meta-analysis intend to fill this gap by assessing the global evidence on the effect of WASH interventions on educational outcomes with due consideration given to gender. This systematic review will include international databases used for the search, such as PubMed, Google Scholar, Web of Science, Europe PubMed Central, and Scopus. Study eligibility will include cross-sectional studies published in English on the impact of WASH interventions on school attendance and academic performance, stratifying gender-specific outcomes. Data extracted will be analyzed using the STATA software version 17. The percentage of heterogeneity will be quantified through the I^2 statistics to show the variability between the included studies. Based on the observed results, diversity will be checked among the outcomes of the study and based on that random-effect model will be used to estimate the pooled effect size. I will, therefore, make use of the Egger and Begg tests for checking statistical asymmetry. Publication bias will be assessed with funnel plots. These will ensure the methodologies used provide comprehensive and rigorous data analysis, which will give strong insights into the impacts of the WASH intervention on educational outcomes.<br>Systematic review and Meta-analysis registration number: PROSPERO CRD42024536477
**Order of Authors:**	Dinaol Bedada Dibaba
	Bezatu Mengistie Alemu, PhD
	Sisay Abebe Debela
**Additional Information:**	
**Question**	**Response**
**Financial Disclosure**<br><br>Enter a financial disclosure statement that describes the sources of funding for the work included in this submission. Review the submission guidelines for detailed	The author(s) received no specific funding for this work.

Powered by Editorial Manager® and ProduXion Manager® from Aries Systems Corporation

requirements. View published research articles from *PLOS ONE* for specific examples.<br><br>This statement is required for submission and **will appear in the published article** if the submission is accepted. Please make sure it is accurate.<br><br>**Funded studies**<br>Enter a statement with the following details:<br>• Initials of the authors who received each award<br>• Grant numbers awarded to each author<br>• The full name of each funder<br>• URL of each funder website<br>• Did the sponsors or funders play any role in the study design, data collection and analysis, decision to publish, or preparation of the manuscript?<br><br>Did you receive funding for this work?	
**Competing Interests**<br><br>Use the instructions below to enter a competing interest statement for this submission. On behalf of all authors, disclose any competing interests that could be perceived to bias this work—acknowledging all financial support and any other relevant financial or non-financial competing interests.<br><br>This statement is required for submission and **will appear in the published article** if the submission is accepted. Please make sure it is accurate and that any funding sources listed in your Funding Information later in the submission form are also declared in your Financial Disclosure statement.<br><br>View published research articles from *PLOS ONE* for specific examples.	The authors have declared that no competing interests exist.

**NO authors have competing interests**<br><br>Enter: *The authors have declared that no competing interests exist.*<br><br>**Authors with competing interests**<br><br>Enter competing interest details beginning with this statement:<br><br>*I have read the journal's policy and the authors of this manuscript have the following competing interests: [insert competing interests here]*<br><br>* typeset	
**Ethics Statement**<br><br>Enter an ethics statement for this submission. This statement is required if the study involved:<br><br>• Human participants<br>• Human specimens or tissue<br>• Vertebrate animals or cephalopods<br>• Vertebrate embryos or tissues<br>• Field research<br><br>Write "N/A" if the submission does not require an ethics statement.<br><br>General guidance is provided below. Consult the submission guidelines for detailed instructions. **Make sure that all information entered here is included in the Methods section of the manuscript.**	N/A

**Format for specific study types**<br><br>**Human Subject Research (involving human participants and/or tissue)**<br>• Give the name of the institutional review board or ethics committee that approved the study<br>• Include the approval number and/or a statement indicating approval of this research<br>• Indicate the form of consent obtained (written/oral) or the reason that consent was not obtained (e.g. the data were analyzed anonymously)<br><br>**Animal Research (involving vertebrate animals, embryos or tissues)**<br>• Provide the name of the Institutional Animal Care and Use Committee (IACUC) or other relevant ethics board that reviewed the study protocol, and indicate whether they approved this research or granted a formal waiver of ethical approval<br>• Include an approval number if one was obtained<br>• If the study involved *non-human primates*, add *additional details* about animal welfare and steps taken to ameliorate suffering<br>• If anesthesia, euthanasia, or any kind of animal sacrifice is part of the study, include briefly which substances and/or methods were applied<br><br>**Field Research**<br><br>Include the following details if this study involves the collection of plant, animal, or other materials from a natural setting:<br>• Field permit number<br>• Name of the institution or relevant body that granted permission	
**Data Availability**<br><br>Authors are required to make the data underlying their research findings fully available, without restriction.<br><br>If the manuscript reports pilot or preliminary results, the data underlying those results must be made available at the time of publication. A Data Availability	N/A - No results are reported

Powered by Editorial Manager® and ProduXion Manager® from Aries Systems Corporation

Statement describing where the data can be found is required at submission. Your answers to this question constitute the Data Availability Statement and will be published in the article, if accepted. When legal or ethical requirements prohibit public data sharing, authors must indicate how researchers may obtain access to the data. See the PLOS Data Policy and FAQ for detailed information.<br><br>**Important:** Stating 'data available on request from the author' is not sufficient. If your data are only available upon request, select 'No' for the first question and explain your situation in the text box.<br><br>For protocols without pilot or preliminary data, authors are strongly encouraged to state how they plan to share research data from their study when it is completed or published.<br><br>If your study includes pilot data, will they be made fully available without restriction?	
**Describe where the data may be found in full sentences. If you are copying our sample text, replace any instances of XXX with the appropriate details.**<br><br>• If **no pilot data are reported** in the manuscript, please enter the following: *No datasets were generated or analysed during the current study. All relevant data from this study will be made available upon study completion.*<br>• If the manuscript reports pilot data **held in a public repository**, include URLs, accession numbers or DOIs. If this information will only be available after acceptance, indicate this by ticking the box below. For example: *All XXX files are available from the XXX database (accession number(s) XXX, XXX.).*<br>• If the manuscript reports pilot data contained **within the manuscript and/or**	No datasets were generated or analysed during the current study. All relevant data from this study will be made available upon study completion.

**Supporting Information files**, enter the following:<br>*All relevant data are within the manuscript and its Supporting Information files.*<br>• If neither of these applies but you are able to provide **details of access elsewhere**, with or without limitations, please do so. For example:<br><br>*Data cannot be shared publicly because of [XXX]. Data are available from the XXX Institutional Data Access / Ethics Committee (contact via XXX) for researchers who meet the criteria for access to confidential data.*<br><br>*The data underlying the results presented in the study are available from (include the name of the third party and contact information or URL).*<br>• This text is appropriate if the data are owned by a third party and authors do not have permission to share the data.<br><br>For study protocols with data management or sharing plans, authors are encouraged to briefly describe in their Data Availability Statement how data they generate will be made accessible when the study is completed. For example:<br>• Deidentified research data will be made publicly available when the study is completed and published.<br><br>* typeset	
Additional data availability information:	

Protocol

Click here to access/download;Manuscript;Updated-Meta Protocol --DINAOL BEDADA.docx

Impact of Water, Sanitation, and Hygiene (WASH) Interventions on Gender-Specific School Attendance and Learning Outcomes: A Systematic Review and Meta-Analysis Protocol

Dinaol Bedada Dibaba[1,2], Bezatu Mengistie Alemu[1], Sisay Abebe Debela[1,2]

[1]Ethiopian Institute of water Resources, Addis Ababa University, Addis Ababa, Ethiopia

[2]Department of Public Health, College of Health Science, Salale University, Fitche, Ethiopia

Corresponding Authors: Dinaol Bedada Dibaba (bedada.dinaol@gmail.com) and Bezatu Mengistie Alemu (bezex2000@yahoo.com)

1. Abstract

The Water, Sanitation, and Hygiene (WASH) interventions have been acknowledged for their role in the public health and educational outcomes. While there are strong evidences that reveal that WASH facilities do reduce the prevalence of infectious diseases and improve the learning environment, data remain thin and equivocal on the differential impacts of WASH facilities on education by gender. The literature reviewed does not, in most cases; go to the extent of investigating if indeed both men and women students have unique needs especially in underprivileged areas. This is the point from which the present systematic review and meta-analysis intend to fill this gap by assessing the global evidence on the effect of WASH interventions on educational outcomes with due consideration given to gender. This systematic review will include international databases used for the search, such as PubMed, Google Scholar, Web of Science, Europe PubMed Central, and Scopus. Study eligibility will include cross-sectional studies published in English on the impact of WASH interventions on school attendance and academic performance, stratifying gender-specific outcomes. Data extracted will be analyzed using the STATA software version 17. The percentage of heterogeneity will be quantified through the I^2 statistics to show the variability between the included studies. Based on the observed results, diversity will be checked among the outcomes of the study and based on that random-effect model will be used to estimate the pooled effect size. I will, therefore, make use of the Egger and Begg tests for checking statistical asymmetry. Publication bias will be assessed with funnel plots. These will ensure the methodologies used provide comprehensive and rigorous data analysis, which will give strong insights into the impacts of the WASH intervention on educational outcomes.

Systematic review and Meta-analysis registration number: PROSPERO **CRD42024536477**

Keywords: WASH interventions, educational outcomes, school attendance.

2. Introduction

Water, Sanitation, and Hygiene (WASH) interventions are key for public health and have a big impact on education and social outcomes all over the world (Chard et al., 2019; Tadesse et al., 2024). Schools especially need good WASH facilities not just for basic cleanliness but also because they help kids come to school more often and boost their ability to learn (Freeman et al., 2012).

It is very important to have effective WASH practices, particularly in places where resources are scarce. Without these, there can be major health and learning problems. Studies have shown that poor WASH setups lead to more sickness and lower school performance, especially in less developed areas (Cairncross et al., 2010; Pruss-Ustun et al., 2014). Take diarrheal diseases, for instance. They are a big reason why kids miss school worldwide, and better sanitation could help reduce this issue (Bowen et al., 2007; Tadesse et al., 2024). In addition, not having proper and safe toilets really affects whether kids especially girls go to school. Girls need these facilities to manage menstrual hygiene, and when they are not there, it hits girls attendance hard (Hennegan et al., 2023; Kansiime et al., 2022; Sommer et al., 2016).

There is evidence that having the right sanitation facilities at school can make a real difference. Schools that have them see their students doing better in their studies compared to schools without them (Alexander et al., 2013; Dreibelbis et al., 2014). This probably comes down to fewer kids getting sick and a better overall environment at school (Chard et al., 2019; Freeman & Clasen, 2011).

On a larger scale, setting up WASH programs in schools contributes to wider economic and social benefits. It helps level the educational playing field and moves society forward (Bartram & Cairncross, 2010; Crocker et al., 2017). These efforts are also part of reaching the United Nations' Sustainable Development Goals, which include targets for health, education, and gender equality (De Guzman et al., 2023; Psaki et al., 2022). But, despite these benefits, the challenge remains huge. Millions of schools around the world still do not have basic WASH facilities, affecting underserved and rural areas hardest and continuing cycles of poverty and poor health (Chakravarty et al., 2017; Wolf et al., 2022). Addressing this issue needs a well-rounded approach that includes installing facilities, educating on hygiene, and getting the community involved (Curtis et al., 2011; Esrey et al., 1991). Adapting efforts to fit the unique cultural and social contexts of communities can make WASH programs more effective (Rheingans et al., 2012).

Despite the recognized benefits of Water, Sanitation, and Hygiene (WASH) interventions, critical gaps exist, particularly in enabling a gender-oriented assessment of their impacts. Most available studies lack gender sensitivity or consideration of socio-cultural factors, which significantly influence the effectiveness of these interventions (Ante-Testard et al., 2024). Additionally, the variability of outcomes across different geographic and socio-economic contexts, influenced by local cultural norms and educational policies, complicates the formulation of effective strategies (Munn et al., 2020; Tadesse et al., 2024). This meta-analysis aims to synthesize global research to elucidate how WASH interventions impact educational outcomes differently for boys and girls, providing precise, actionable insights that can inform policy and enhance educational equity through tailored WASH practices.

3. Review Question:

I. How do WASH interventions influence school attendance rates, specifically analyzing differences based on gender?

II. What is the effect of WASH interventions on learning outcomes and academic performance across genders?

III. Do the effects of WASH interventions on educational outcomes demonstrate variability across different geographic regions?

4. Methodology

4.1.Search Strategy:

For this systematic review and meta-analysis, different search strategies were employed including but not limited to general search string and database-specific search string such as PubMed, PubMed Central, Scopus, Web of Science and Cochrane Library.

4.1.1 General search string

("water, sanitation, and hygiene" OR "WASH interventions" OR "sanitation facilities" OR "hygiene facilities") AND ("school attendance" OR "academic performance" OR "learning outcomes" OR "educational outcomes").

4.1.2. Database-Specific Search Strings

a) PubMed

For PubMed free text and Mesh terms, (((((((("impact"[Title/Abstract] OR "effect"[Title/Abstract]) AND "water"[Title/Abstract]) OR "water"[MeSH Terms]) AND

"sanitation"[Title/Abstract]) OR "sanitation"[MeSH Terms]) AND "hygiene"[Title/Abstract]) OR "hygiene"[MeSH Terms]) AND "School"[Title/Abstract]) OR "schools"[MeSH Terms]) AND "attendance"[Title/Abstract] search queries were used.

PubMed free text searches, ((((impact[tiab]) OR (effect[tiab])) AND ((((water[tiab]) AND (sanitation[tiab])) AND (hygiene[tiab])) OR (WASH[tiab]))) AND (School[tiab])) AND (attendance[tiab]) search queries were used.

b) Scopus

For Scopus which allows more flexible text word searches, TITLE-ABS-KEY ("Impact of water, sanitation, and hygiene" OR "effect of water, sanitation, and hygiene" OR "effect of WASH" OR "impact of WASH" OR "effect of sanitation facilities" OR "impact of sanitation facilities" OR "effect of hygiene" OR "impact of hygiene") AND ("school attendance" OR "academic performance" OR "learning outcomes") search queries were used.

c) Web of Science

Web of Science search interface using Boolean operators and nested queries, TS=((" water, sanitation, and hygiene" OR " WASH interventions" OR " sanitation facilities" OR "impact of hygiene facilities") AND (" school attendance" OR "academic performance" OR "learning outcomes")) search queries were used.

d) Cochrane Library

For Cochrane Library, ("water, sanitation, and hygiene" OR "WASH interventions" OR "sanitation" OR "hygiene") AND ("school attendance" OR "learning outcomes" OR "academic achievement") search queries were used.

4.2. Screening Strategy

Titles and abstracts will be independently screened to assess potential relevance based on the inclusion and exclusion criteria.

4.3. Inclusion And Exclusion Criteria

4.3.1. Inclusion Criteria:

Study Types: Institutional cross-sectional studies reporting primary data.

Population: School-aged children (ages 6-18) attending formal educational institutions.

Interventions: Studies evaluating the impact of WASH interventions.

Outcomes: Studies must report gender-specific educational outcomes such as differences in school attendance or academic performance between male and female students.

Data Source: Both published and unpublished studies done in English available globally will be considered.

4.3.2. Exclusion Criteria:

Studies that do not provide data disaggregated by gender, and studies not in educational settings.

4.4. Data Extraction And Reporting:

To ensure uniformity and free from biases when extracting data from all of the studies included in this review, We will use standardized data extraction forms. The form has been designed to capture details of information that are critical in understanding the scope and impacts of WASH interventions. From each of the studies, We will extract the following:

Author(s) and Year of Publication: For citation and reference tracking.

Country of Study: To evaluate geographical diversity and contextual applicability.

Study Design: This encompasses the design of the studies undertaken.

Population Details: Demographic description of the study population, such as the age and gender distribution.

Education Outcome: Include data for schooling attendance of education outcome and academic achievement outcome, paying special focus on gender disparity.

Sample Size: Number of participants, which is critical for statistical analyses. Statistical **Measures:** Key statistics such as effect sizes, confidence intervals, and p-values.

4.5. Data Analysis

To ascertain the level of heterogeneity among the included studies on the impact of Water, Sanitation, and Hygiene (WASH) interventions on gender-specific educational outcomes, the I^2 statistic will be utilized, with values over 75% indicating substantial heterogeneity. This is a common approach in educational and social sciences meta-analysis (Borenstein et al., 2010). Publication bias will be assessed through Egger's regression test and visual examination of funnel plots, techniques endorsed for their efficacy in social science research (Sterne et al., 2011). All statistical analyses will be rigorously conducted using STATA version 17, facilitating a comprehensive and precise synthesis of data aimed at enhancing educational and public health strategies.

4.6.Sensitivity Analysis:

In this protocol, sensitivity analysis is systematically applied to ensure the reliability of the findings regarding the effects of Water, Sanitation and Hygiene (WASH) interventions on educational outcomes. This methodological approach involves testing the stability of the results under various conditions, such as using different statistical models (fixed-effect vs. random-effect) and altering the inclusion criteria to exclude studies with higher risks of bias or lower quality. By doing so, We aim to identify whether the conclusions drawn are sensitive to the chosen methods or specific subsets of data. This thorough examination helps validate the robustness of the outcomes, enhancing the study's credibility and providing clear, evidence-based insights that can guide policy-making and educational program enhancements. Additionally, sensitivity analysis promotes transparency in the research process, allowing for a deeper understanding of how methodological choices influences the study's conclusions.

4.7.Critical Appraisal:

Critical Appraisal will be systematically employed to ensure the integrity and validity of the included studies. This will be encouraged through the application of standard tools, such as Newcastle-Ottawa Scale for the observational studies.

4.8.Limitations:

Several potential limitations are anticipated in this meta-analysis. One of the concerns is the variability in how WASH interventions are implemented across different settings, which might affect the comparability of results. Additionally, there is a risk of publication bias, as studies showing significant or positive results are more likely to be published than those that do not, potentially skewing the overall findings. Given the diverse settings of the studies involved, there may also be substantial heterogeneity, which could complicate the interpretation of the pooled estimates. In some cases, relevant data may be incomplete or missing, limiting the analysis of certain outcomes. Finally, the impact of socio-cultural variables, which could influence the effectiveness of WASH interventions, might be underrepresented due to the lack of detailed reporting in the original studies.

4.9.Operational definitions:

WASH Interventions: Initiatives aimed at improving water quality, sanitation facilities, and hygiene practices within school settings. These interventions are intended to enhance

public health and educational outcomes by providing cleaner environments and reducing disease transmission.

School Attendance: The regular presence of school-aged children (ages 6-18) in formal educational institutions.

Academic Performance: The educational outcomes and achievements of students in their schoolwork.

Learning Outcomes: While term summarizes the broader cognitive, emotional, and social competencies acquired by students through schooling. Learning outcomes in this document are evaluated in relation to the presence and quality of WASH facilities.

Gender-Specific Educational Outcomes: These are the differences in educational results between male and female students, particularly focusing on how WASH interventions may affect each gender differently, such as through facilities that cater to menstrual hygiene management that primarily affects female students.

Statistical Measures (Effect Sizes, Confidence Intervals, p-values): Used to describe the magnitude and significance of the effects observed in the study. These include the size of the impact WASH interventions have on gender-specific educational outcomes, the reliability of these estimates, and the statistical significance of the results.

Heterogeneity (I^2 statistic): A statistical measure used to quantify the variance among the included studies. Values over 75% indicate substantial heterogeneity, affecting the interpretation of pooled data in meta-analyses.

Publication Bias (Funnel Plots, Egger's Regression Test): Techniques used to assess asymmetry in meta-analysis data, which can indicate whether the synthesis of results might be biased by the non-publication of negative or inconclusive studies.

4.10. Quality assessment and study selection process:

For the quality assessment of the studies included in our systematic review and meta-analysis on the effects of Water Sanitation and Hygiene (WASH) interventions on educational outcomes, two authors (Dinaol Bedada and Bezatu Mengistie) will independently evaluate each study using the Joanna Briggs Institute (JBI) (McKenzie & Brennan, 2019; Page et al., 2020) critical appraisal

checklist appropriate for the respective study designs. Each study will be scored on a scale from 0 to 100%, and only those receiving a score of 50% or higher will be included in further analysis (Shea et al., 2017). In cases where there are discrepancies between the assessments, DBD and BMA will discuss to resolve differences or, if necessary, involve a third, impartial reviewer to achieve consensus. The results of this quality assessment is indicated in a PRISMA flow diagram (Figure1) (Page et al., 2021), which will detail the study selection process, including the number of studies screened, assessed for eligibility, included in the review, and excluded with reasons at each stage. This approach ensures the reliability and validity of the included studies in our meta-analysis.

4.11. Data Extraction form

See “Data extraction form –DINAOL.docx” file

5. DISCUSSION:

Significant gaps exist in the current literature regarding the differential impacts of Water Sanitation and Hygiene (WASH) interventions on educational outcomes for students, particularly in disadvantaged regions. These disparities often go unaddressed, highlighting the need for a systematic review and meta-analysis. By focusing on gender-specific educational outcomes, this systematic review and Meta-Analysis aims to provide a detailed understanding of how these interventions can meet the unique needs of different student groups, thereby enhancing their effectiveness and inclusivity.

Our research approach involved search strategy across several databases to ensure a thorough evaluation of available data. We executed detailed searches in PubMed, Web of Science, Scopus, and the Cochrane Library, retrieving a many records: 1677 from PubMed, 27 from Web of Science, 7 from Scopus, and 67 from the Cochrane Library. Our strategy employed both free text and controlled vocabulary terms to maximize the capture of relevant studies, highlighting the scale and depth of our investigation. This methodological approach is intended to capture a wide range of studies, ensuring a detailed analysis of the available evidence.

In synthesizing this evidence, we expect to fill the significant research gaps by providing robust, differentiated insights into the effectiveness of WASH interventions. This will not only contribute to the academic literature but also offer actionable guidance for policymakers and educational authorities. By contextualizing the potential outcomes within the scope of public health and

educational enhancement, we aim to foster a more inclusive and effective approach to implementing WASH programs globally. This protocol sets the stage for a comprehensive exploration of WASH interventions, paving the way for future studies and interventions that are informed by gender-sensitive research and practice.

Supporting Information:

The included supporting files with this protocol are: "Data extraction form –DINAOL.docx", "Search Strategies used--DINAOL.docx", "PRISMA-P Protocol checklist --DINAOL.docx", and "PRISMA_2020_flow_diagram--DINAOL.docx".

Authors Contribution:

Conceptualizations, data curation, formal analysis, editing, validation, writing original draft, writing reviewing and methodology development performed by Dinaol Bedada Dibaba and Bezatu Mengistie Alemu while Bezatu Mengistie Alemu performed supervision. Additionally Sisay Abebe Debela participated in editing, revision and analysis.

Ethical approval and consent to participate:

Not applicable.

Consent for publication:

Not applicable.

Conflict of interests:

The authors declare that they have no competing interests.

Funding:

No funding obtained for this systematic review and Meta-Analysis protocol

References

Alexander, K. T., Dreibelbis, R., Freeman, M. C., Ojeny, B., & Rheingans, R. (2013). Improving service delivery of water, sanitation, and hygiene in primary schools: a cluster-randomized trial in western Kenya. *J Water Health*, *11*(3), 507–519. https://doi.org/10.2166/wh.2013.213

Ante-Testard, P. A., Rerolle, F., Nguyen, A. T., Ashraf, S., Parvez, S. M., Naser, A. M., Benmarhnia, T., Rahman, M., Luby, S. P., Benjamin-Chung, J., & Arnold, B. F. (2024). WASH interventions and child diarrhea at the interface of climate and socioeconomic

position in Bangladesh. *Nat Commun*, *15*(1), 1556. https://doi.org/10.1038/s41467-024-45624-1

Bartram, J., & Cairncross, S. (2010). Hygiene, sanitation, and water: forgotten foundations of health. *PLoS Med*, *7*(11), e1000367. https://doi.org/10.1371/journal.pmed.1000367

Borenstein, M., Hedges, L. V., Higgins, J. P. T., & Rothstein, H. R. (2010). A basic introduction to fixed-effect and random-effects models for meta-analysis. *Research Synthesis Methods*, *1*(2), 97–111. https://doi.org/10.1002/JRSM.12

Bowen, A., Ma, H., Ou, J., Billhimer, W., Long, T., Mintz, E., Hoekstra, R. M., & Luby, S. (2007). A cluster-randomized controlled trial evaluating the effect of a handwashing-promotion program in Chinese primary schools. *Am J Trop Med Hyg*, *76*(6), 1166–1173. https://www.ncbi.nlm.nih.gov/pubmed/17556631

Cairncross, S., Bartram, J., Cumming, O., & Brocklehurst, C. (2010). Hygiene, Sanitation, and Water: What Needs to Be Done? *Plos Medicine*, *7*(11). https://doi.org/ARTN e1000365 10.1371/journal.pmed.1000365

Chakravarty, I., Bhattacharya, A., & Das, S. K. (2017). Water, sanitation and hygiene: The unfinished agenda in the World Health Organization South-East Asia Region. *WHO South East Asia J Public Health*, *6*(2), 22–33. https://doi.org/10.4103/2224-3151.213787

Chard, A. N., Garn, J. V, Chang, H. H., Clasen, T., & Freeman, M. C. (2019). Impact of a school-based water, sanitation, and hygiene intervention on school absence, diarrhea, respiratory infection, and soil-transmitted helminths: results from the WASH HELPS cluster-randomized trial. *J Glob Health*, *9*(2), 20402. https://doi.org/10.7189/jogh.09.020402

Crocker, J., Saywell, D., & Bartram, J. (2017). Sustainability of community-led total sanitation outcomes: Evidence from Ethiopia and Ghana. *Int J Hyg Environ Health*, *220*(3), 551–557. https://doi.org/10.1016/j.ijheh.2017.02.011

Curtis, V., Schmidt, W., Luby, S., Florez, R., Toure, O., & Biran, A. (2011). Hygiene: new hopes, new horizons. *Lancet Infect Dis*, *11*(4), 312–321. https://doi.org/10.1016/S1473-3099(10)70224-3

De Guzman, K., Stone, G., Yang, A. R., Schaffer, K. E., Lo, S., Kojok, R., Kirkpatrick, C. R., Del Pozo, A. G., Le, T. T., DePledge, L., Frost, E. L., & Kayser, G. L. (2023). Drinking water and the implications for gender equity and empowerment: A systematic review of qualitative and quantitative evidence. *Int J Hyg Environ Health*, *247*, 114044. https://doi.org/10.1016/j.ijheh.2022.114044

Dreibelbis, R., Freeman, M. C., Greene, L. E., Saboori, S., & Rheingans, R. (2014). The impact of school water, sanitation, and hygiene interventions on the health of younger siblings of pupils: a cluster-randomized trial in Kenya. *Am J Public Health*, *104*(1), e91-7. https://doi.org/10.2105/AJPH.2013.301412

Esrey, S. A., Potash, J. B., Roberts, L., & Shiff, C. (1991). Effects of improved water supply and sanitation on ascariasis, diarrhoea, dracunculiasis, hookworm infection, schistosomiasis, and trachoma. *Bull World Health Organ*, *69*(5), 609–621. https://www.ncbi.nlm.nih.gov/pubmed/1835675

Freeman, M. C., & Clasen, T. (2011). Assessing the impact of a school-based safe water intervention on household adoption of point-of-use water treatment practices in southern India. *Am J Trop Med Hyg*, *84*(3), 370–378. https://doi.org/10.4269/ajtmh.2011.10-0361

Freeman, M. C., Greene, L. E., Dreibelbis, R., Saboori, S., Muga, R., Brumback, B., & Rheingans, R. (2012). Assessing the impact of a school-based water treatment, hygiene and sanitation programme on pupil absence in Nyanza Province, Kenya: a cluster-randomized trial. *Trop Med Int Health*, *17*(3), 380–391. https://doi.org/10.1111/j.1365-3156.2011.02927.x

Hennegan, J., Caruso, B. A., Zulaika, G., Torondel, B., Haver, J., Phillips-Howard, P. A., Valdez, J., Gruer, C., Okwaro, N., Mahon, T., & Sommer, M. (2023). Indicators for National and Global Monitoring of Girls' Menstrual Health and Hygiene: Development of a Priority Shortlist. *J Adolesc Health*, *73*(6), 992–1001. https://doi.org/10.1016/j.jadohealth.2023.07.017

Kansiime, C., Hytti, L., Nelson, K. A., Torondel, B., Francis, S. C., Tanton, C., Greco, G., Belfield, S., Nakalema, S., Matovu, F., Ssemata, A. S., Alezuyo, C., Neema, S., Jerrim, J., Bonell, C., Seeley, J., Weiss, H. A., & Group, M. (2022). Menstrual health interventions, schooling, and mental health problems among Ugandan students (MENISCUS): study protocol for a school-based cluster-randomised trial. *Trials*, *23*(1), 759. https://doi.org/10.1186/s13063-022-06672-4

McKenzie, J. E., & Brennan, S. E. (2019). Cochrane Handbook for Systematic Reviews of Interventions. In *Cochrane Handbook for Systematic Reviews of Interventions* (pp. 321–347). wiley. https://doi.org/10.1002/9781119536604.ch12

Munn, Z., Tufanaru, C., Lockwood, C., Stern, C., McAneney, H., & Barker, T. H. (2020). Rinse-free hand wash for reducing absenteeism among preschool and school children. *Cochrane Database Syst Rev*, *4*(4), CD012566. https://doi.org/10.1002/14651858.CD012566.pub2

Page, M. J., McKenzie, J. E., Bossuyt, P. M., Boutron, I., Hoffmann, T. C., Mulrow, C. D., Shamseer, L., Tetzlaff, J. M., Akl, E. A., Brennan, S. E., Chou, R., Glanville, J., Grimshaw, J. M., Hróbjartsson, A., Lalu, M. M., Li, T., Loder, E. W., Mayo-Wilson, E., McDonald, S., … Moher, D. (2021). The PRISMA 2020 statement: an updated guideline for reporting systematic reviews. *BMJ*, *372*. https://doi.org/10.1136/BMJ.N71

Page, M. J., McKenzie, J. E., Bossuyt, P. M., Boutron, I., Hoffmann, T., Mulrow, C. D., Shamseer, L., & Moher, D. (2020). Mapping of reporting guidance for systematic reviews and meta-analyses generated a comprehensive item bank for future reporting guidelines. *J Clin Epidemiol*, *118*, 60–68. https://doi.org/10.1016/j.jclinepi.2019.11.010

Pruss-Ustun, A., Bartram, J., Clasen, T., Colford Jr., J. M., Cumming, O., Curtis, V., Bonjour, S., Dangour, A. D., De France, J., Fewtrell, L., Freeman, M. C., Gordon, B., Hunter, P. R., Johnston, R. B., Mathers, C., Mausezahl, D., Medlicott, K., Neira, M., Stocks, M., … Cairncross, S. (2014). Burden of disease from inadequate water, sanitation and hygiene in low- and middle-income settings: a retrospective analysis of data from 145 countries. *Trop Med Int Health*, *19*(8), 894–905. https://doi.org/10.1111/tmi.12329

Psaki, S., Haberland, N., Mensch, B., Woyczynski, L., & Chuang, E. (2022). Policies and interventions to remove gender-related barriers to girls' school participation and learning in low- and middle-income countries: A systematic review of the evidence. *Campbell Syst Rev*, *18*(1), e1207. https://doi.org/10.1002/cl2.1207

Rheingans, R., Kukla, M., Faruque, A. S., Sur, D., Zaidi, A. K., Nasrin, D., Farag, T. H., Levine, M. M., & Kotloff, K. L. (2012). Determinants of household costs associated with childhood diarrhea in 3 South Asian settings. *Clin Infect Dis*, *55 Suppl 4*(Suppl 4), S327-35. https://doi.org/10.1093/cid/cis764

Shea, B. J., Reeves, B. C., Wells, G., Thuku, M., Hamel, C., Moran, J., Moher, D., Tugwell, P., Welch, V., Kristjansson, E., & Henry, D. A. (2017). AMSTAR 2: a critical appraisal tool for systematic reviews that include randomised or non-randomised studies of healthcare interventions, or both. *BMJ*, *358*. https://doi.org/10.1136/bmj.j4008

Sommer, M., Caruso, B. A., Sahin, M., Calderon, T., Cavill, S., Mahon, T., & Phillips-Howard, P. A. (2016). A Time for Global Action: Addressing Girls' Menstrual Hygiene Management Needs in Schools. *PLoS Med*, *13*(2), e1001962. https://doi.org/10.1371/journal.pmed.1001962

Sterne, J. A. C., Sutton, A. J., Ioannidis, J. P. A., Terrin, N., Jones, D. R., Lau, J., Carpenter, J., Rücker, G., Harbord, R. M., Schmid, C. H., Tetzlaff, J., Deeks, J. J., Peters, J., Macaskill, P., Schwarzer, G., Duval, S., Altman, D. G., Moher, D., & Higgins, J. P. T. (2011). Recommendations for examining and interpreting funnel plot asymmetry in meta-analyses of randomised controlled trials. *BMJ*, *343*(7818). https://doi.org/10.1136/BMJ.D4002

Tadesse, G., Wuletaw, Y., Mekete, K., Sime, H., Yard, E., Appleby, L., Grimes, J., Dejene, N., Gardiner, I., Kazienga, A., Abbeddou, S., French, M., Levecke, B., & Drake, L. (2024). Investigating the effect of a school-based WASH intervention on soil-transmitted helminth and schistosome infections and nutritional status of school children in Ethiopia: a quasi-experimental study. *Parasit Vectors*, *17*(1), 130. https://doi.org/10.1186/s13071-024-06155-2

Wolf, J., Hubbard, S., Brauer, M., Ambelu, A., Arnold, B. F., Bain, R., Bauza, V., Brown, J., Caruso, B. A., Clasen, T., Colford Jr., J. M., Freeman, M. C., Gordon, B., Johnston, R. B., Mertens, A., Pruss-Ustun, A., Ross, I., Stanaway, J., Zhao, J. T., … Boisson, S. (2022). Effectiveness of interventions to improve drinking water, sanitation, and handwashing with soap on risk of diarrhoeal disease in children in low-income and

middle-income settings: a systematic review and meta-analysis. *Lancet*, *400*(10345), 48–59. https://doi.org/10.1016/S0140-6736(22)00937-0

Click here to access/download
Supporting Information
Search Strategies used--DINAOL.docx

PRISMA protocol checklist

Click here to access/download

Supporting Information

PRISMA-P Protocol checklist --DINAOL.docx

PRISMA Flow diagram

Click here to access/download
Supporting Information
PRISMA_2020_flow_diagram--DINAOL.docx

Data extraction form

Click here to access/download

Supporting Information

Data extraction form --DINAOL.docx
